# Potential Ophthalmological Side Effects Induced by Anti-Neoplastic Regimens for the Treatment of Genitourinary Cancers: A Review

**DOI:** 10.7759/cureus.27266

**Published:** 2022-07-26

**Authors:** Minas Sakellakis, Nikolaos Spathas, Konstantinos T Tsaousis, Emmanouil N Nikitiadis, Helena Linardou, Vasilios F Diakonis

**Affiliations:** 1 Medical Oncology, Hellenic Genitourinary Cancer Group, Athens, GRC; 2 Fourth Oncology Department and Comprehensive Clinical Trial Center, Metropolitan Hospital, Athens, GRC; 3 Ophthalmology, Volos General Hospital, Volos, GRC; 4 Department of Internal Medicine, Metropolitan Hospital, Athens, GRC; 5 Second Ophthalmology Clinic, Metropolitan Hospital, Athens, GRC

**Keywords:** cancers, genitourinary, effects, adverse, ophthalmologic

## Abstract

The outcomes of patients with genitourinary (GU) cancers have been steadily improving in recent years. Novel therapies have entered our armamentarium, while several other regimens are currently being studied in clinical trials. This recent explosion of new agents has improved patient survival and the quality of life for patients, but has also significantly increased the frequency of several side effects. The current review will focus on the potential ocular adverse reactions of GU neoplastic treatments. The broad spectrum of manifestations of ocular toxicity underscores the uniqueness and complexity of the anatomic, physiologic, and metabolic features of the human eye. Most side effects are mild in severity and transient, but some can be severe, disabling, and irreversible. Clinicians should be aware of complications that might be vision threatening and impact the patient's quality of life. In this review, we focused on the ocular toxicity of the antineoplastic regimens that are currently used for the treatment of GU, including prostate cancer, bladder cancer, renal cell carcinoma, testicular cancer, pheochromocytoma, adrenocortical carcinoma, and penile cancer.

## Introduction and background

The outcomes of patients with genitourinary (GU) cancers have been steadily improving in recent years. Novel therapies have entered our armamentarium, while several other regimens are currently being studied in clinical trials. In prostate cancer, the addition of second-generation antiandrogens increased the effectiveness of androgen ablation and led to increased patient survival [1,2]. Molecular targeted therapies have also recently entered clinical practice in prostate cancer [3]. In bladder cancer, the development of immune checkpoint inhibitors, drug-antibody conjugates, and FGFR (fibroblast growth factor receptor) inhibitors has dramatically transformed the therapeutic landscape [4-6]. The introduction of immune checkpoint inhibitors and novel tyrosine kinase inhibitors into clinical practice has also revolutionized the treatment of patients with metastatic renal cell carcinoma [7,8]. This recent explosion of new regimens has improved patient survival, but it has also significantly increased the frequency of reported cases of ocular side effects [9,10]. This can be attributed not only to the growing number of used agents but also due to the fact that patients demonstrate a longer life expectancy. Unfortunately, underreporting regarding ocular side effects exists and the majority of the available literature is provided from case reports [9-11]. Interestingly, many molecular targets of the novel GU anti-neoplastic regimens are expressed in the eye, which may have implications and demonstrate several side effects in the ocular and periocular tissues [12]. The broad spectrum of manifestations of ocular toxicity underscores the complexity and uniqueness of the anatomic, physiologic, and metabolic features of the human eye. Most side effects are mild in severity and transient, but some can be severe, disabling, and irreversible [11,12]. Clinicians should be aware of complications that might be vision-threatening. Ophthalmologists need to be familiar with the side effects of a growing number of regimens that enter clinical practice. Newer agents have distinct mechanisms of action compared to classical chemotherapeutics. Finally, combinations of antitumor regimens might make it difficult to attribute a side effect to a specific drug. Knowledge of the mechanism underlying specific ocular side effects is sometimes critical for proper management. Here, we will focus on the ocular toxicity of the antineoplastic regimens that are currently used for the treatment of GU cancers, including prostate cancer, bladder cancer, renal cell carcinoma, testicular cancer, pheochromocytoma, adrenocortical carcinoma, and penile cancer.

## Review

Androgen deprivation therapy

Androgen deprivation remains the mainstay of therapy for patients with hormone-sensitive prostate cancer. Pharmaceutical castration has been traditionally achieved with the use of either luteinizing hormone-releasing hormone (LHRH) receptor agonists, such as leuprolide, goserelin, and triptorelin, or antagonists, such as degarelix, ganirelix, and abarelix [13]. Since their introduction into clinical practice, antiandrogens have dramatically changed the natural history of the disease. However, they are associated with a multitude of systemic side effects that can impact the quality of life [14].

Chronic androgen deficiency is linked to meibomian gland dysfunction and dry eye syndrome [15] (Table 1). Studies have shown that antiandrogens alter the relative amounts of lipids in the Meibomian gland secretions [16]. As a result, scientists observed increased tear film debris, irregular posterior lid margins, abnormal tear film meniscus, decreased tear film break-up time, and orifice metaplasia of the Meibomian glands. The decreased quality of meibomian gland secretions is associated with increased light sensitivity, blurred vision, foreign body sensation, and painful eyes [15,16].

**Table 1:** Ophthalmological effects of anti-neoplastic regimens used in the treatment of prostate cancer. Ophthalmological adverse effects and potential beneficial effects of antitumor agents used in prostate cancer management. Molecular markers in prostate cancer that serve as targets for newer antineoplastic agents, also exist in the human eye. This results not only in ocular side effects, but also in effects that might prove to be useful in the treatment of different ophthalmic conditions. LHRH: luteinizing hormone-releasing hormone, BRVO: branch retinal vein occlusion, PSMA: prostate specific membrane antigen, PARP: poly adenosine diphosphate-ribose polymerase.

Medications	Side effects (frequency)	Beneficial potential
Antiandrogens: LHRH agonists/antagonists (long-term administration)	Dry eye syndrome (frequent), tear film debris (28.6%), irregular lid margin (85.7%), tear film mucous (10.3%), abnormal tear film meniscus (40%), light sensitivity, blurry vision, ocular pain (all together 50%), optic neuritis, cataract development, BRVO, retinal hemorrhages (all rare)	None reported
Second generation antiandrogens: enzalutamide, abiraterone	Blurry vision (rare)	None reported
Chemotherapy: docetaxel	Canalicular stenosis and nasolacrimal duct obstruction: epiphora (7.9%), cystoid macular edema, erosive conjunctivitis, ocular pain, decreased vision, scintillating scotomas, toxic optic neuropathy, uveal effusions (all rare)	None reported
Chemotherapy: cabazitaxel	Optic neuropathy, decreased vision, color vision deficiency, visual field defects (all rare)	None reported
Theranostics: PSMA	Dry eye syndrome (30%)	Angiogenesis inhibitor, treatment of angiogenesis based ocular conditions
PARP inhibitors: Olaparib, Rucaparib, Niraparib	Conjunctivitis (6%), eyelid swelling (unknown), decreased vision (unknown)	Protective role in age-related dry macular degeneration, treatment of photoreceptors in hereditary retinal dystrophies or glaucoma, protective of cataract development

Androgen-deprivation therapy may be accompanied by headaches and dizziness after each drug injection in about half of the cases, and may also cause a transient decrease in vision (Table 1). These symptoms usually last less than one to two hours, but they can sometimes persist for more than three to four weeks [17,18]. Although robust cause-and-effect relationship data are lacking, antiandrogens have also been linked to pseudotumor cerebri, cataracts, and optic neuritis [19-22].

It has been suggested that androgen deprivation can also result in thromboembolic phenomena, intraocular branch vein occlusion, and hemorrhages [23-25]. However, a recent population-based cohort study found no evidence supporting an increased risk of retinal vascular occlusion [25]. Second-generation antiandrogens include androgen receptor inhibitors, such as enzalutamide, apalutamide, and darolutamide, as well as CYP17A1 inhibitors, such as abiraterone. Enzalutamide (and other second-generation antiandrogens) frequently causes blurred vision, which is usually self-limiting. In rare cases, enzalutamide can cause PRES (posterior reversible encephalopathy) syndrome with headache, seizures, impaired vision, and hypertension [26,27]. Although definite evidence is lacking, enzalutamide-mediated GABA receptor inhibition in the central nervous system (CNS) has been suggested as a potential mechanism [27].

Chemotherapy

Cisplatin is a chemotherapeutic regimen (alkylating agent) that is widely used in the treatment of bladder cancer, testicular cancer, penile cancer, and adrenocortical carcinoma. It is also occasionally used in patients with aggressive castration-resistant prostate cancer [6,28-30]. Cisplatin is occasionally associated with ischemic retinopathy and neovascularization, while the co-administration of other chemotherapeutics might have a synergistic effect [31] (Table 2). Cisplatin has also been associated with painless color vision changes, granular pigmentary deposits, papilledema, optic neuritis, retrobulbar neuritis, transient cortical blindness, temporary homonymous hemianopia, and bilateral central scotomas [32-36].

**Table 2:** Ophthalmological effects of anti-neoplastic regimens used in the treatment of bladder cancer. Adverse effects and potential beneficial effects of anti-neoplastic regimens that are currently used in the treatment of bladder cancer. While older chemotherapeutics in regular concentrations rarely cause ocular toxicity, newer classes of agents, such as drug-antibody conjugates or FGFR inhibitors are marked by a high incidence of ocular toxicity. Immune checkpoint inhibitors cause ocular side effects in less than 1% of cases. ICIs: immune checkpoint inhibitors, FGFR: fibroblast growth factor receptor.

Medications	Side effects (frequency)	Beneficial potential
Chemotherapy: Cisplatin	Color vision changes (dose-dependent/unknown), granular pigmentary deposits (rare), optic neuritis, retrobulbar neuritis, transient cortical blindness, temporary homonymous hemianopia, bilateral central scotomas (all dose-dependent/rare in regular doses), ischemic retinopathy (rare), neovascularization (rare)	None reported
Chemotherapy: Carboplatin	Blurred vision, eye soreness, chorioretinitis, optic neuritis, papilledema (all rare)	None reported
Chemotherapy: Paclitaxel	Transient scintillating scotomas (20%), optic nerve edema (rare)	None reported
Chemotherapy: Methotrexate	Anterior surface irritation (46% in high dose), periorbital edema, ocular pain, dry eye, blurry vision, photophobia, blepharitis, conjunctivitis, decreased reflex tear secretions (all together up to 25% in high dose), optic neuritis (rare)	None reported
Chemotherapy: Doxorubicin	Conjunctivitis, excessive lacrimation, periorbital edema, blepharospasm, keratitis, decreased visual acuity (all rare)	None reported
Immune checkpoint inhibitors: Avelumab	Uveitis, iritis (all together <1%)	None reported
ICI: Durvalumab	Uveitis, iritis, keratitis (all together <1%)	None reported
Drug-antibody conjugates: Enfortumab vedotin	Dry eye (23%), blurry vision (15%), excessive lacrimation (14%), keratitis, limbal stem cell deficiency (unknown) (all together up to 40%)	None reported
Drug-antibody conjugates: Sacituzumab govitecan	Periorbital edema (unknown)	None reported
Tyrosine kinase inhibitors: Erdafitinib	Central serous retinopathy, dry eyes, conjunctivitis, increased lacrimation, blurry vision, cataracts, keratitis, and corneal erosions (all together up to 21%)	None reported

Carboplatin is another alkylating agent that is used in patients with bladder cancer, testicular cancer, and adrenocortical carcinoma. Carboplatin can rarely cause blurred vision, eye soreness, chorioretinitis, or optic neuritis [11].

Oxaliplatin is used in patients with chemotherapy-resistant testicular cancer [28]. Ocular symptoms associated with oxaliplatin include abnormal lacrimation, blepharoptosis, conjunctivitis, blurry vision, tunnel vision, and color perception abnormalities. Rare cases of uveitis, keratitis, iritis, cataracts, retinal damage, and visual field loss have also been reported [12,37].

Ifosfamide (a cyclophosphamide analog) is another alkylating agent which is frequently used in patients with testicular, bladder, and penile cancer [6,28,30]. It has been associated with reversible blurry vision and conjunctivitis [11].

Docetaxel is currently the first-line chemotherapy regimen for patients with metastatic prostate cancer (Table 1). Docetaxel is a member of taxanes and inhibits mitosis in dividing cancer cells by preventing microtubule depolymerization and attenuating the effects of bcl-2 and bcl-xL expression [38,39]. It frequently causes canalicular and nasolacrimal duct obstruction, with resulting epiphora [40,41]. Docetaxel is secreted in the tear film, which results in canalicular chronic inflammation and is thought to be the cause of canalicular stenosis. Epiphora may interfere with daily life activities and consists a troublesome symptom for patients. The use of lubricant eye drops may help wash out docetaxel and prevent dacryostenosis [40,41]. Docetaxel also rarely causes cystoid macular edema and erosive conjunctivitis [40,42,43]. Common symptoms include redness of the eye, pain and itchiness, decreased vision, and scintillating scotomas [42,43]. A few reports have also linked docetaxel administration with uvela effusions, outer retinal disruption, and toxic optic neuropathy [44,45].

Paclitaxel is another taxane frequently used in patients with bladder cancer, testicular cancer, and penile cancer. In approximately 20% of patients, paclitaxel causes transient scintillating scotomas [46,47]. These symptoms usually appear near the end of the infusion and last 15 minutes to 3 hours [11,48,49]. It is unclear whether they are caused by neurotoxicity or transient ischemia. Prolonged optic nerve edema is a rare but potentially vision-threatening side effect of paclitaxel. The combination of paclitaxel with cisplatin can synergistically increase the neurotoxicity risk to the optic nerve [49]. Patients receiving the above chemotherapeutic combination can present with blepharitis, conjunctivitis, visual field defects, periorbital edema, cystoid macular edema, retinal pigment disturbances, altered color perception (cone dysfunction), and mild retinal ischemic changes (cotton-wool spots, posterior pole intraretinal hemorrhage) [49,50].

Another taxane frequently used in prostate cancer, cabazitaxel, has been associated with blurred vision, color vision deficiency, and visual field defects as a result of optic neuropathy [51,52].

Vincristine and other plant alkaloids occasionally cause reversible cranial nerve palsies (upper lid ptosis, lagophthalmos, corneal hypoesthesia). The median time of onset of these symptoms is 10 weeks after initiating treatment. Vincristine is also reported to cause optic neuropathy and even transient cortical blindness in high doses [11]. The chemotherapeutic combination of vincristine-cyclophosphamide-dacarbazine for the treatment of pheochromocytoma has been associated with reversible blurred vision, blepharoconjunctivitis, keratoconjunctivitis sicca, and pinpoint pupils [11,53]. However, pheochromocytoma itself can be the cause of hypertensive retinopathy, choroidopathy, and optic neuropathy [54,55].

Etoposide is a vinca alkaloid, which is frequently used in the treatment of patients with testicular cancer and adrenocortical carcinoma [28,29]. Rare case reports have linked etoposide with retinal toxicity [56].

Methotrexate is an antimetabolite chemotherapeutic regimen that is commonly used in patients with bladder cancer [57]. At high doses, it is often associated with mild anterior ocular surface irritation a few days after the beginning of treatment [58]. Methotrexate has been associated with periorbital edema, ocular pain, dry eye, blurred vision, photophobia, blepharitis, conjunctivitis, and decreased reflex tear secretions [59]. Longstanding methotrexate use is rarely associated with optic neuropathy [60]. Common symptoms include visual field scotomas, optic nerve edema, and optic atrophy [61,62]. It is believed that methotrexate-induced optic neuropathy is linked to folate deficiency since folate supplementation reduces the severity of the condition [63].

Fluoropyrimidines are antimetabolites frequently used in patients with penile and bladder cancer. They are also used in patients with bladder cancer who undergo bladder preservation chemoradiation [6,30]. Fluoropyrimidines are sometimes associated with excessive tearing caused by ocular surface disease, canalicular stenosis, dry eye syndrome, and nasolacrimal system obstruction [11,12,64-66]. Other symptoms include ocular pain, blurred vision, eye irritation, photophobia, irritative conjunctivitis, circumorbital edema, and keratitis. These symptoms usually resolve one to two weeks after treatment cessation [11,12]. Eyelid conjunctival hyperemia, lid margin abnormalities, corneal erosions, and lower eyelid punctal edema are common findings [64,65]. Under specific circumstances, 5-FU can cause eyelid ectropion, eyelid margin fusion, as well as neuro-ophthalmologic toxicity [11,67].

Doxorubicin is an anthracycline frequently used in bladder cancer patients and occasionally in patients with adrenocortical carcinoma [6,29]. It is associated with conjunctivitis, especially in conjunction with docetaxel, cyclophosphamide, and fluorouracil [68]. Doxorubicin is also associated with excessive lacrimation, periorbital edema, blepharospasm, keratitis, and decreased visual acuity. Most adverse reactions usually resolve within 24 hours after exposure [69].

Streptozotocin is an alkylating agent useful in subsets of patients with adrenocortical carcinoma [70]. Streptozotocin damages pancreatic beta cells in preclinical models and can potentially induce diabetes mellitus [71]. However, there is evidence that human beta cells are resistant to the diabetogenic effects of streptozotocin [72]. Ocular side effects are rare, but a small percentage of patients report conjunctivitis and excessive lacrimation [11].

Mitomycin-C is an antitumor antibiotic agent that is frequently used in penile and bladder cancer patients [6,30]. While topical mitomycin-C has been widely used in corneal refractive surgery, its systemic use has been mostly associated with blurry vision [11,73,74].

Mitotane is a steroidogenesis inhibitor and exerts cytotoxic effects on the adrenal cortex. Mitotane, which is a widely used agent in the treatment of adrenocortical carcinoma, is the only adrenal-specific chemotherapeutic regimen [29,75]. It has been associated with visual blurring, cataracts, visual diplopia, edema, toxic retinopathy with retinal hemorrhage, papilloedema, and retinal pigmentary changes [76].

Immune checkpoint inhibitors

The introduction of immune checkpoint inhibitors (ICIs) has recently transformed the management of bladder cancer and renal cell carcinoma [77]. Under specific circumstances, ICIs are useful in the treatment of most GU malignancies [78]. Due to non-specific immune activation, they are able to cause immune-related side effects in almost every system. Ocular side effects are rare and reported in 1-3% of patients that are treated with ICIs [79] (Table 3). Fang et al. reported an increased incidence of uveitis and other eye inflammatory conditions with the use of PD-1 inhibitors nivolumab and pembrolizumab. ICI-induced uveitis can occur with or without hypotony [80]. Patients receiving nivolumab or pembrolizumab had an increased risk of dry eyes and ocular myasthenia [80]. Cases of keratitis, thyroid-like orbitopathy, retinal vasculitis, orbital neuropathy, and choroiditis have been reported in the literature [81].

**Table 3:** Ophthalmological effects of anti-neoplastic regimens used in the treatment of kidney cancer. Ophthalmological adverse effects and potentially beneficial effects of anti-neoplastic agents that are currently used in the treatment of kidney cancer. Apart from periorbital edema, ocular side effects of tyrosine kinase inhibitors are generally rare. Ocular toxicity rates of immune checkpoint inhibitors in the treatment of renal cell carcinoma range from 1% to 3% (except tearing that occurs in about 9%) of cases. Newer hypoxia-inducible factor inhibitors and mTOR inhibitors are marked by a relatively high rate of ocular toxicity. TKIs are being studied as potential local treatments of ocular neo-angiogenesis. TKIs: tyrosine kinase inhibitors, mTOR: mammalian target of rapamycin.

Medications	Side effects (frequency)	Beneficial potential
TKIs: Sunitinib, pazopanib, sorafenib	Periorbital edema (15.9% for sunitinib), reversible posterior leukoencephalopathy syndrome, blurred vision, eyelid or periocular edema, superficial anterior segment toxicity, and conjunctival, vitreous or retinal bleeding, extraocular muscle disorders, eyelash discoloration, retinal venous or arterial occlusions, papilledema, ischemic optic neuropathy, macular edema, uveitis, retinal detachment or retinal tears (all rare)	None reported
TKIs: Axitinib, cabozantinib, lenvatinib	Retinal vein thrombosis, retinal toxicity, reversible posterior leukoencephalopathy syndrome (for lenvatinib) (all rare)	Potential local treatments of ocular neo-angiogenesis
Immune checkpoint inhibitors: Nivolumab, pembrolizumab	Uveitis, dry eye, ocular myasthenia, keratitis, thyroid-like orbitopathy, retinal vasculitis, orbital neuropathy, choroiditis (all together up to 1-3%)	None reported
Immune checkpoint inhibitors: Ipilimumab	Tearing (9%), uveitis (<1%), dry eye (<1%)	None reported
Hypoxia-inducible factor inhibitors: Belzutifan	Blurry vision, retinal detachment, central retinal vein occlusion (all together up to 21%)	None reported
mTOR inhibitors: Everolimus	Eyelid edema (1-10%), conjunctivitis (1-10%)	None reported
mTOR inhibitors: Temsirolimus	Conjunctivitis (1-10%), tearing (1-10%), eyelid edema (rare)	None reported

Avelumab and durvalumab are PD-L1 inhibitors commonly used in bladder cancer [77]. Ocular side effects are less common with PD-L1 inhibitors, compared to PD-1 inhibitors [80,82]. In fewer than 1% of cases, avelumab and durvalumab are associated with uveitis and iritis. Durvalumab has also been linked to keratitis, while Andrade et al. reported a case of durvalumab-induced retinal vasculitis [80,82].

Ipilimumab is a CTLA-4 inhibitor commonly used in patients with metastatic renal cell carcinoma [77]. Uveitis and other inflammatory ocular side effects, along with dry eyes, have been associated with ipilimumab use in cancer patients. Excessive lacrimation has also been reported [80,83,84]. Although the ocular toxicity of PD-1 inhibitors is not dose-dependent, ipilimumab toxicity is dose-dependent [85,86]. Therefore, ipilimumab use may be accompanied by a lower incidence of ocular adverse effects.

Tyrosine kinase inhibitors

Sunitinib, pazopanib, and sorafenib belong to the family of tyrosine kinase inhibitors. They have been widely used in the treatment of advanced renal cell carcinoma [7]. They inhibit several receptor tyrosine kinases, including VEGFR, platelet-derived growth factor receptor (PDGFR), and others [87]. Sunitinib, pazopanib, and sorafenib can cause RPLS with disturbance of cerebral vascular autoregulation, which eventually leads to breakdown of the blood-brain barrier [88,89] (Table 3). This side effect is often related to hypertension due to disruptions in renal autoregulation. Ocular side effects develop due to visual cortex edema. Adequate treatment should be promptly applied (i.e., blood pressure reduction) in order to avoid long-term ocular complications, such as centrocaecal scotoma [89]. RPLS has also been reported after bevacizumab treatment [89]. It has been suggested that bevacizumab-induced vasospasm may trigger the syndrome and result in sometimes not fully reversible cortical blindness [89].

Fraunfelder et al. reported that the most common ocular side effects in patients receiving oral sunitinib, pazopanib, and sorafenib included blurry vision, eyelid or periocular edema, superficial anterior segment toxicity, and conjunctival, vitreous, or retinal bleeding [90]. Other side effects included extraocular muscle disorders, eyelash discoloration, retinal venous or arterial occlusions, papilledema, ischemic optic neuropathy, macular edema, and uveitis [90]. Importantly, these agents have been linked to serious cases of retinal detachment or retinal tears [90].

Axitinib, cabozantinib, and lenvatinib are potent TKIs that recently entered our armamentarium in renal cell carcinoma, alone or in combination with immune checkpoint inhibitors [91]. Due to their inhibitory effect on VEGFR 1, 2, and 3 signaling, they are being evaluated experimentally as potential local treatments for ocular neo-angiogenesis [92]. However, ocular side effects from systemic use are rare. There have been reports associating systemic axitinib administration with microangiopathic retinal toxicity [93,94]. There is also a potential association between cabozantinib use and the development of bilateral optic disc edema [94,95]. Lenvatinib has been rarely linked to reversible posterior leukoencephalopathy syndrome (RPLS) [96].

FGFR inhibitors are a new class of antineoplastic drugs that are currently being tested in several clinical trials for various malignancies [97]. Erdafitinib is an FGFR inhibitor that has shown activity against advanced bladder cancers, which harbor specific FGFR mutations [6,97]. Eye disorders are frequently reported during treatment with erdafitinib [98]. However, most patients are able to continue treatment [98] (Table 2). Similar to other FGFR inhibitors, erdafitinib has been associated with central serous retinopathy [99]. This results in fluid accumulation under the retina, causing retinal epithelium detachment and central blurry vision [100]. The condition resembles MEK inhibitor-induced retinopathy, possibly because FGFR and MEK pathways intersect [100]. Patients can be asymptomatic if the affected area falls outside the macula. The condition is usually self-limited and vision recovery occurs within one to four months. However, if the condition recurs or persists for more than four to six months, surgical interventions, such as photodynamic therapy or subthreshold micropulse laser treatment, might be necessary [101]. Erdafitinib has also been associated with dry eyes, conjunctivitis, increased lacrimation, blurry vision, cataracts, keratitis, and corneal erosions [101].

Drug-antibody conjugates

An antibody-drug conjugate is a monoclonal antibody that targets an antigen, which is specific for a particular tumor, conjugated with a cytotoxic agent. Enfortumab vedotin (directed against nectin-4) and sacituzumab govitecan (directed against the human trophoblast cell-surface antigen 2) have shown activity and can confer a survival advantage in the treatment of metastatic refractory bladder cancer [6,102]. However, they appear to be particularly toxic to the cornea. Enfortumab vedotin is frequently associated with dry eye, keratitis, blurry vision, excessive lacrimation, and limbal stem cell deficiency [103,104] (Table 2). Ocular toxicity of sacituzumab govitecan appears to be less frequently reported, although periorbital edema is a well-known side effect [105].

PARP inhibitors

PARP (poly-ADP-ribose polymerase) inhibitors, such as olaparib, rucaparib, and niraparib, have been recently added to the prostate cancer therapy armamentarium since they were shown to prolong overall survival in patients with metastatic castration-resistant prostate cancer (mCRPC) [106] (Table 1). PARP1-dependent cell death in the retinal pigment epithelium has also been implicated in age-related macular degeneration (AMD), while studies have shown that PARP inhibitors might exert a protective role in dry AMD [107]. PARP inhibitors might also be beneficial in the treatment of photoreceptor degeneration in hereditary retinal dystrophies or glaucoma [108,109]. Moreover, preclinical studies suggest that PARP inhibitors have a protective role against factors contributing to the development of age- or diabetes-related cataract [110,111]. A common ophthalmic side effect in patients receiving PARP inhibitors is conjunctivitis. Patients might also experience eyelid swelling or blurred vision [112-114].

Theranostics

PSMA (prostate-specific membrane antigen) ligands are rapidly entering the diagnostic and therapeutic landscape of prostate cancer (Table 1). PSMA is also expressed in the lacrimal glands, which results in dry eyes in 30-40% of patients who are exposed to PSMA-targeted radioligands [115,116].

mTOR inhibitors

Temsirolimus and everolimus are inhibitors of the mammalian target of rapamycin (mTOR) kinase, which is a component of a signaling pathway involved in cellular proliferation and response to hypoxic stress. The disruption of mTOR signaling inhibits cell cycle progression and angiogenesis [117]. The most common ocular side effects of everolimus are eyelid edema and conjunctivitis [94,118] (Table 3). Frequently, everolimus-induced eyelid edema might need to be treated surgically [118]. Conjunctivitis, including lacrimation disorder, is the most common ophthalmic side effect of temsirolimus. Temsirolimus can also rarely result in eyelid edema [94].

Hypoxia-inducible factor inhibitors

Hypoxia-inducible factor (HIF) inhibitors constitute a new promising class of drugs for the treatment of advanced renal cell carcinoma [7,119]. HIF-2alpha is closely related to retinal hypoxia, and HIF-2alpha inhibitors have shown promise for neovascularization inhibition in oxygen-induced retinopathy mouse models [120]. Belzutifan is a novel HIF-2alpha inhibitor, currently used against von Hippel-Lindau disease-associated RCC [119]. Ocular side effects of belzutifan are very common, with visual impairment appearing in roughly 21% of patients. Frequent findings include blurry vision, retinal detachment, and central retinal vein occlusion [121] (Table 3).

Somatostatin analogs

Somatostatin analogs are widely used in the treatment of pheochromocytoma. They are known to have edema-reducing effects in patients with macular edema [122,123]. Somatostatin is synthesized in the retina, and somatostatin receptors are present in retinal pigment epithelial cells [124]. Experimental intravitreal injections of somatostatin-analogs (octreotide) have shown the potential to halt retinal neovascularization [125]. Furthermore, studies investigate their potential role against the development of proliferative vitreoretinopathy and diabetic neovascularization [125,126]. Rare side effects of somatostatin analogs include visual field defects and increased intraocular pressure.

Interferons

Interferon in combination with bevacizumab is sometimes used in the treatment of metastatic renal cell carcinoma [7,127]. Interferon administration is associated with retinopathy, which can present with cotton wool spots, retinal hemorrhage, optic disc swelling, micro-aneurysms, macular thickening, or edema [128].

EGFR inhibitors

Cetuximab is an epidermal growth factor receptor (EGFR) inhibitor frequently used in the treatment of penile cancer [30,129]. Cetuximab is most commonly associated with conjunctivitis, eyelash trichomegaly, eyelid dermatitis, blepharitis, corneal erosions, and keratitis [130-132].

## Conclusions

Given the life-threatening nature of several cancer-related complications, ocular side effects are often underestimated and underreported. A baseline ophthalmic examination might be useful in detecting pre-existing conditions, and it can help decrease the rate of significant and irreversible ocular toxicity. Healthcare professionals should also be encouraged to report ocular side effects. In conclusion, oncologists and ophthalmologists should work together to counter the ocular adverse outcomes of anticancer treatments, which can lead to significant visual disturbance and impact the patient's quality of life.

## References

[1] James ND, de Bono JS, Spears MR (2017). Abiraterone for prostate cancer not previously treated with hormone therapy. N Engl J Med.

[2] Chi KN, Agarwal N, Bjartell A (2019). Apalutamide for metastatic, castration-sensitive prostate cancer. N Engl J Med.

[3] de Bono J, Mateo J, Fizazi K (2020). Olaparib for metastatic castration-resistant prostate cancer. N Engl J Med.

[4] Powles T, Rosenberg JE, Sonpavde GP (2021). Enfortumab vedotin in previously treated advanced urothelial carcinoma. N Engl J Med.

[5] Powles T, Park SH, Voog E (2020). Avelumab maintenance therapy for advanced or metastatic urothelial carcinoma. N Engl J Med.

[6] Peng M, Xiao D, Bu Y, Long J, Yang X, Lv S, Yang X (2020). Novel combination therapies for the treatment of bladder cancer. Front Oncol.

[7] Kathuria-Prakash N, Drolen C, Hannigan CA, Drakaki A (2021). Immunotherapy and metastatic renal cell carcinoma: a review of new treatment approaches. Life (Basel).

[8] Rini BI, Plimack ER, Stus V (2019). Pembrolizumab plus Axitinib versus Sunitinib for Advanced Renal-Cell Carcinoma. N Engl J Med.

[9] Vishnevskia-Dai V, Rozner L, Berger R, Jaron Z, Elyashiv S, Markel G, Zloto O (2021). Ocular side effects of novel anti-cancer biological therapies. Sci Rep.

[10] Schmid KE, Kornek GV, Scheithauer W, Binder S (2006). Update on ocular complications of systemic cancer chemotherapy. Surv Ophthalmol.

[11] al-Tweigeri T, Nabholtz JM, Mackey JR (1996). Ocular toxicity and cancer chemotherapy. A review. Cancer.

[12] Singh P, Singh A (2012). Ocular adverse effects of anti-cancer chemotherapy. J Cancer Ther Res.

[13] Crawford ED, Heidenreich A, Lawrentschuk N (2019). Androgen-targeted therapy in men with prostate cancer: evolving practice and future considerations. Prostate Cancer Prostatic Dis.

[14] Ziółkowska E, Zarzycka M, Wiśniewski T, Zyromska A (2012). The side effects of hormonal therapy at the patients with prostate cancer. Contemp Oncol (Pozn).

[15] Krenzer KL, Dana MR, Ullman MD (2000). Effect of androgen deficiency on the human meibomian gland and ocular surface. J Clin Endocrinol Metab.

[16] Sullivan BD, Evans JE, Krenzer KL, Reza Dana M, Sullivan DA (2000). Impact of antiandrogen treatment on the fatty acid profile of neutral lipids in human meibomian gland secretions. J Clin Endocrinol Metab.

[17] Fraunfelder FT, Edwards R (1995). Possible ocular adverse effects associated with leuprolide injections. JAMA.

[18] Bolton EM, Lynch T (2018). Are all gonadotrophin-releasing hormone agonists equivalent for the treatment of prostate cancer? A systematic review. BJU Int.

[19] Omar AA, Nyaga G, Mungai LN (2020). Pseudotumor cerebri in patient on leuprolide acetate for central precocious puberty. Int J Pediatr Endocrinol.

[20] Boot JH (1996). Pseudotumour cerebri as a side effect of leuprorelin acetate. Ir J Med Sci.

[21] Beebe-Dimmer J, Morgenstern H, Cetin K (2011). Androgen deprivation therapy and cataract incidence among elderly prostate cancer patients in the United States. Ann Epidemiol.

[22] Ní Mhéalóid Á, Cunniffe G (2017). Optic neuritis secondary to antiandrogen therapy. Ir J Med Sci.

[23] Federici TJ (2007). Leuprolide acetate and central retinal vein occlusion. Ophthalmic Surg Lasers Imaging.

[24] Zaoui M, Cordebar B, Naoun-Hubert I, Sommer S, Rozot P (2000). Occlusion de la veine centrale de la rétine sous anti-androgènes [Central retinal vein occlusion in a patient treated with antiandrogenic drug]. J Fr Ophtalmol.

[25] Lin HL, Lee CY, Huang JY, Tseng PC, Yang SF (2022). Androgen deprivation therapy for prostate cancer did not increase the risk of retinal vascular occlusion: a population-based cohort study. Int J Environ Res Public Health.

[26] (2022). United States Prescribing Information for enzalutamide available. https://www.accessdata.fda.gov/drugsatfda_docs/label/2018/203415s014lbl.pdf.

[27] Crona DJ, Whang YE (2015). Posterior reversible encephalopathy syndrome induced by enzalutamide in a patient with castration-resistant prostate cancer. Invest New Drugs.

[28] Feldman DR, Bosl GJ, Sheinfeld J, Motzer RJ (2008). Medical treatment of advanced testicular cancer. JAMA.

[29] Kiesewetter B, Riss P, Scheuba C, Mazal P, Kretschmer-Chott E, Haug A, Raderer M (2021). Management of adrenocortical carcinoma: are we making progress?. Ther Adv Med Oncol.

[30] Stecca CE, Alt M, Jiang DM, Chung P, Crook JM, Kulkarni GS, Sridhar SS (2021). Recent advances in the management of penile cancer: a contemporary review of the literature. Oncol Ther.

[31] Kwan AS, Sahu A, Palexes G (2006). Retinal ischemia with neovascularization in cisplatin related retinal toxicity. Am J Ophthalmol.

[32] Martin M, Weber-Várszegi J, Flammer J (2005). [Toxic optic neuropathy due to cisplatin therapy: a case report]. Klin Monbl Augenheilkd.

[33] Fischer N, Stuermer J, Rodic B, Pless M (2009). Carboplatin-induced bilateral papilledema: a case report. Case Rep Oncol.

[34] Miller DF, Bay JW, Lederman RJ, Purvis JD, Rogers LR, Tomsak RL (1985). Ocular and orbital toxicity following intracarotid injection of BCNU (carmustine) and cisplatinum for malignant gliomas. Ophthalmology.

[35] Caraceni A, Martini C, Spatti G, Thomas A, Onofrj M (1997). Recovering optic neuritis during systemic cisplatin and carboplatin chemotherapy. Acta Neurol Scand.

[36] (1990). Cisplatin neurotoxicity. N Engl J Med.

[37] Noor A, Desai A, Singh M (2019). Reversible ocular toxicity of oxaliplatin: a case report. Cureus.

[38] de Morrée ES, Vogelzang NJ, Petrylak DP (2017). Association of survival benefit with docetaxel in prostate cancer and total number of cycles administered: a post hoc analysis of the mainsail study. JAMA Oncol.

[39] Pienta KJ (2001). Preclinical mechanisms of action of docetaxel and docetaxel combinations in prostate cancer. Semin Oncol.

[40] Noguchi Y, Kawashima Y, Maruyama M, Kawara H, Tokuyama Y, Uchiyama K, Shimizu Y (2020). Current status of eye disorders caused by docetaxel administration every 3 weeks: a case-control study in Japanese patients. J Oncol Pharm Pract.

[41] Yamagishi T, Ochi N, Yamane H, Hasebe S, Takigawa N (2014). Epiphora in lung cancer patients receiving docetaxel: a case series. BMC Res Notes.

[42] Enzsoly A, Kammerer K, Nemeth J, Schneider M (2015). Bilateral cystoid macular edema following docetaxel chemotherapy in a patient with retinitis pigmentosa: a case report. BMC Ophthalmol.

[43] Chalvatzis N, Manthou ME, Tzamalis A, Hytiroglou P, Dimitrakos S (2014). Erosive conjunctival and corneal inflammatory changes in a patient receiving weekly docetaxel for breast cancer. Ocul Immunol Inflamm.

[44] Moloney TP, Xu W, Rallah-Baker K, Oliveira N, Woodward N, Farrah JJ (2014). Toxic optic neuropathy in the setting of docetaxel chemotherapy: a case report. BMC Ophthalmol.

[45] Kord Valeshabad A, Mieler WF, Setlur V, Thomas M, Shahidi M (2015). Posterior segment toxicity after gemcitabine and docetaxel chemotherapy. Optom Vis Sci.

[46] Gianni L, Munzone E, Capri G (1995). Paclitaxel in metastatic breast cancer: a trial of two doses by a 3-hour infusion in patients with disease recurrence after prior therapy with anthracyclines. J Natl Cancer Inst.

[47] Scaioli V, Caraceni A, Martini C, Curzi S, Capri G, Luca G (2006). Electrophysiological evaluation of visual pathways in paclitaxel-treated patients. J Neurooncol.

[48] Seidman AD, Barrett S, Canezo S (1994). Photopsia during 3-hour paclitaxel administration at doses &gt; or = 250 mg/m2. J Clin Oncol.

[49] Li Y, Li Y, Li J, Pi G, Tan W (2014). Paclitaxel- and/or cisplatin-induced ocular neurotoxicity: a case report and literature review. Onco Targets Ther.

[50] Noguchi Y, Kawashima Y, Maruyama M, Kawara H, Tokuyama Y, Uchiyama K, Shimizu Y (2018). Risk factors for eye disorders caused by paclitaxel: a retrospective study. Biol Pharm Bull.

[51] Noguchi Y, Kawashima Y, Kawara H, Kaneko M, Nakauchi H, Tokuyama Y (2016). [An undeniable case of optic neuropathy due to cabazitaxel]. Gan To Kagaku Ryoho.

[52] Diker S, Diker Ö (2019). Optic atrophy after cabazitaxel treatment in a patient with castration-resistant prostate cancer: a case report. Scott Med J.

[53] Niemeijer ND, Alblas G, van Hulsteijn LT, Dekkers OM, Corssmit EP (2014). Chemotherapy with cyclophosphamide, vincristine and dacarbazine for malignant paraganglioma and pheochromocytoma: systematic review and meta-analysis. Clin Endocrinol (Oxf).

[54] Petkou D, Petropoulos IK, Kordelou A, Katsimpris JM (2008). Severe bilateral hypertensive retinopathy and optic neuropathy in a patient with pheochromocytoma. Klin Monbl Augenheilkd.

[55] Matsubara N, Kato A, Kominami A, Nozaki M, Yasukawa T, Yoshida M, Ogura Y (2019). Bilateral giant retinal pigment epithelial tears in hypertensive choroidopathy. Am J Ophthalmol Case Rep.

[56] Das A, Ranjan R, Das N, Shah PK (2020). Bilateral macular ischemia following oral etoposide. Indian J Ophthalmol.

[57] Droller MJ (2000). Neoadjuvant cisplatin, methotrexate, and vinblastine chemotherapy for muscle-invasive bladder cancer: a randomised controlled trial. J Urol.

[58] Doroshow JH, Locker GY, Gaasterland DE, Hubbard SP, Young RC, Myers CE (1981). Ocular irritation from high-dose methotrexate therapy: pharmacokinetics of drug in the tear film. Cancer.

[59] Peponis V, Kyttaris VC, Chalkiadakis SE, Bonovas S, Sitaras NM (2010). Ocular side effects of anti-rheumatic medications: what a rheumatologist should know. Lupus.

[60] Balachandran C, McCluskey PJ, Champion GD, Halmagyi GM (2002). Methotrexate-induced optic neuropathy. Clin Exp Ophthalmol.

[61] Sbeity ZH, Baydoun L, Schmidt S (2006). Visual field changes in methotrexate therapy. Case report and review of the literature. J Med Liban.

[62] Johansson BA (1992). Visual field defects during low-dose methotrexate therapy. Doc Ophthalmol.

[63] Clare G, Colley S, Kennett R, Elston JS (2005). Reversible optic neuropathy associated with low-dose methotrexate therapy. J Neuroophthalmol.

[64] Eiseman AS, Flanagan JC, Brooks AB, Mitchell EP, Pemberton CH (2003). Ocular surface, ocular adnexal, and lacrimal complications associated with the use of systemic 5-fluorouracil. Ophthalmic Plast Reconstr Surg.

[65] Bonadonna G, Brusamolino E, Valagussa P (1976). Combination chemotherapy as an adjuvant treatment in operable breast cancer. N Engl J Med.

[66] Prasad S, Kamath GG, Phillips RP (2000). Lacrimal canalicular stenosis associated with systemic 5-fluorouacil therapy. Acta Ophthalmol Scand.

[67] Insler MS, Helm CJ (1987). Ankyloblepharon associated with systemic 5-fluorouracil treatment. Ann Ophthalmol.

[68] Karamitsos A, Kokkas V, Goulas A, Paraskevopoulos P, Gougoulias K, Karampatakis V, Boboridis K (2013). Ocular surface and tear film abnormalities in women under adjuvant chemotherapy for breast cancer with the 5-Fluorouracil, Epirubicin and Cyclophosphamide (FEC) regimen. Hippokratia.

[69] Curran CF, Luce JK (1989). Ocular adverse reactions associated with adriamycin (doxorubicin). Am J Ophthalmol.

[70] Alyateem G, Nilubol N (2021). Current status and future targeted therapy in adrenocortical cancer. Front Endocrinol (Lausanne).

[71] Furman BL (2015). Streptozotocin-induced diabetic models in mice and rats. Curr Protoc Pharmacol.

[72] Yang H, Wright JR Jr (2002). Human beta cells are exceedingly resistant to streptozotocin in vivo. Endocrinology.

[73] Diakonis VF, Pallikaris A, Kymionis GD, Markomanolakis MM (2007). Alterations in endothelial cell density after photorefractive keratectomy with adjuvant mitomycin. Am J Ophthalmol.

[74] Arranz-Marquez E, Katsanos A, Kozobolis VP, Konstas AG, Teus MA (2019). A critical overview of the biological effects of mitomycin c application on the cornea following refractive surgery. Adv Ther.

[75] Shariq OA, McKenzie TJ (2021). Adrenocortical carcinoma: current state of the art, ongoing controversies, and future directions in diagnosis and treatment. Ther Adv Chronic Dis.

[76] Ng WT, Toohey MG, Mulhall L, Mackey DA (2003). Pigmentary retinopathy, macular oedema, and abnormal ERG with mitotane treatment. Br J Ophthalmol.

[77] Parikh M, Powles T (2021). Immune checkpoint inhibition in advanced bladder and kidney cancer: responses and further management. Am Soc Clin Oncol Educ Book.

[78] Twomey JD, Zhang B (2021). Cancer immunotherapy update: FDA-approved checkpoint inhibitors and companion diagnostics. AAPS J.

[79] Shahzad O, Thompson N, Clare G, Welsh S, Damato E, Corrie P (2021). Ocular adverse events associated with immune checkpoint inhibitors: a novel multidisciplinary management algorithm. Ther Adv Med Oncol.

[80] Fang T, Maberley DA, Etminan M (2019). Ocular adverse events with immune checkpoint inhibitors. J Curr Ophthalmol.

[81] Nguyen M, Islam MR, Lim SW, Sahu A, Tamjid B (2019). Pembrolizumab induced ocular hypotony with near complete vision loss, interstitial pulmonary fibrosis and arthritis. Front Oncol.

[82] R Andrade A, Moll-Udina A, Martin R, Cilveti E, Subirà O, Disfetano L, García-Arumí J (2020). Retinal vasculitis secondary to durvalumab. Case Rep Ophthalmol.

[83] McElnea E, Ní Mhéalóid A, Moran S, Kelly R, Fulcher T (2014). Thyroid-like ophthalmopathy in a euthyroid patient receiving Ipilimumab. Orbit.

[84] Robinson MR, Chan CC, Yang JC (2004). Cytotoxic T lymphocyte-associated antigen 4 blockade in patients with metastatic melanoma: a new cause of uveitis. J Immunother.

[85] Attia P, Phan GQ, Maker AV (2005). Autoimmunity correlates with tumor regression in patients with metastatic melanoma treated with anti-cytotoxic T-lymphocyte antigen-4. J Clin Oncol.

[86] Martins F, Sofiya L, Sykiotis GP (2019). Adverse effects of immune-checkpoint inhibitors: epidemiology, management and surveillance. Nat Rev Clin Oncol.

[87] Alonso-Gordoa T, García-Bermejo ML, Grande E, Garrido P, Carrato A, Molina-Cerrillo J (2019). Targeting tyrosine kinases in renal cell carcinoma: "new bullets against old guys". Int J Mol Sci.

[88] Kapiteijn E, Brand A, Kroep J, Gelderblom H (2007). Sunitinib induced hypertension, thrombotic microangiopathy and reversible posterior leukencephalopathy syndrome. Ann Oncol.

[89] Hager T, Seitz B (2013). Ocular side effects of biological agents in oncology: what should the clinician be aware of?. Onco Targets Ther.

[90] Fraunfelder FT, Fraunfelder FW (2018). Oral anti-vascular endothelial growth factor drugs and ocular adverse events. J Ocul Pharmacol Ther.

[91] Rassy E, Flippot R, Albiges L (2020). Tyrosine kinase inhibitors and immunotherapy combinations in renal cell carcinoma. Ther Adv Med Oncol.

[92] Giddabasappa A, Lalwani K, Norberg R (2016). Axitinib inhibits retinal and choroidal neovascularization in in vitro and in vivo models. Exp Eye Res.

[93] Jenkins TL, Aderman CM, Ho AC (2021). Reversible retinal toxicity in a patient taking axitinib. Retin Cases Brief Rep.

[94] Huillard O, Bakalian S, Levy C (2014). Ocular adverse events of molecularly targeted agents approved in solid tumours: a systematic review. Eur J Cancer.

[95] Huang YT, Lin CJ, Tsai YY, Hsia NY (2022). Bilateral optic disc edema as a possible complication of cabozantinib use-a case report. Eur J Ophthalmol.

[96] Osawa Y, Gozawa R, Koyama K, Nakayama T, Sagoh T, Sunaga H (2018). Posterior reversible encephalopathy syndrome after lenvatinib therapy in a patient with anaplastic thyroid carcinoma. Intern Med.

[97] Kommalapati A, Tella SH, Borad M, Javle M, Mahipal A (2021). FGFR inhibitors in oncology: insight on the management of toxicities in clinical practice. Cancers (Basel).

[98] Loriot Y, Necchi A, Park SH (2019). Erdafitinib in locally advanced or metastatic urothelial carcinoma. N Engl J Med.

[99] Claiborne RT, Tsan GL (2022). Case report: erdafitinib-induced central serous chorioretinopathy. Optom Vis Sci.

[100] Fasolino G, Moschetta L, De Grève J, Nelis P, Lefesvre P, Ten Tusscher M (2022). Choroidal and choriocapillaris morphology in Pan-FGFR inhibitor-associated retinopathy: a case report. Diagnostics (Basel).

[101] (2022). Canadian prescribing information for enfortumab erdafitinib. https://www.janssenmedicalinformation.ca/sites/www.janssenmedicalinformation.ca/files/JOI-BAL.pdf?v=436.

[102] Vlachostergios PJ, Jakubowski CD, Niaz MJ (2018). Antibody-drug conjugates in bladder cancer. Bladder Cancer.

[103] Rosenberg JE, O'Donnell PH, Balar AV (2019). Pivotal trial of enfortumab vedotin in urothelial carcinoma after platinum and anti-programmed death 1/programmed death ligand 1 therapy. J Clin Oncol.

[104] (2022). United States prescribing information for enfortumab vedotin. https://www.accessdata.fda.gov/drugsatfda_docs/label/2019/761137s000lbl.pdf.

[105] (2022). United States Prescribing Information for sacituzumab govitecan. https://www.accessdata.fda.gov/drugsatfda_docs/label/2020/761115s000lbl.pdf.

[106] Grewal K, Grewal K, Tabbara IA (2021). PARP inhibitors in prostate cancer. Anticancer Res.

[107] Ho J, Jang KH, Koo TS, Park C, Kim YH, Lee J, Kim E (2021). Protective effects of PARP1-inhibitory compound in dry age-related macular degeneration. Biomed Pharmacother.

[108] Sahaboglu A, Sharif A, Feng L, Secer E, Zrenner E, Paquet-Durand F (2017). Temporal progression of PARP activity in the Prph2 mutant rd2 mouse: neuroprotective effects of the PARP inhibitor PJ34. PLoS One.

[109] Mohanty K, Dada R, Dada T (2017). Oxidative DNA damage and reduced expression of DNA repair genes: role in primary open angle glaucoma (POAG). Ophthalmic Genet.

[110] Singh A, Bodakhe SH (2021). Biochemical evidence indicates the preventive effect of resveratrol and nicotinamide in the treatment of STZ-induced diabetic cataract. Curr Eye Res.

[111] Cencer CS, Chintala SK, Townsend TJ (2018). PARP-1/PAR activity in cultured human lens epithelial cells exposed to two levels of UVB light. Photochem Photobiol.

[112] Butler SK (2018). Niraparib (Zejula). Oncol Times.

[113] Pratt G, Yap C, Oldreive C (2018). A multi-centre phase I trial of the PARP inhibitor olaparib in patients with relapsed chronic lymphocytic leukaemia, T-prolymphocytic leukaemia or mantle cell lymphoma. Br J Haematol.

[114] (2022). United States prescribing information for olaparib. https://www.accessdata.fda.gov/drugsatfda_docs/label/2018/208558s001lbl.pdf.

[115] Chandran E, Figg WD, Madan R (2022). Lutetium-177-PSMA-617: a vision of the future. Cancer Biol Ther.

[116] Klein Nulent TJ, Valstar MH, de Keizer B (2018). Physiologic distribution of PSMA-ligand in salivary glands and seromucous glands of the head and neck on PET/CT. Oral Surg Oral Med Oral Pathol Oral Radiol.

[117] Faes S, Demartines N, Dormond O (2021). Mechanistic target of rapamycin inhibitors in renal cell carcinoma: potential, limitations, and perspectives. Front Cell Dev Biol.

[118] Schear MJ, Rodgers R (2018). A case of everolimus-induced eyelid edema. Ophthalmic Plast Reconstr Surg.

[119] Jonasch E, Donskov F, Iliopoulos O (2021). Belzutifan for renal cell carcinoma in von Hippel-Lindau disease. N Engl J Med.

[120] Zhang J, Qin Y, Martinez M (2021). HIF-1α and HIF-2α redundantly promote retinal neovascularization in patients with ischemic retinal disease. J Clin Invest.

[121] (2022). United States prescribing information for belzutifan. https://www.accessdata.fda.gov/drugsatfda_docs/label/2021/215383s000lbl.pdf.

[122] Leijon H, Remes S, Hagström J (2019). Variable somatostatin receptor subtype expression in 151 primary pheochromocytomas and paragangliomas. Hum Pathol.

[123] Kafkala C, Choi JY, Choopong P, Foster CS (2006). Octreotide as a treatment for uveitic cystoid macular edema. Arch Ophthalmol.

[124] Hernández C, Simó-Servat O, Simó R (2014). Somatostatin and diabetic retinopathy: current concepts and new therapeutic perspectives. Endocrine.

[125] Evren O, Turgut B, Celiker U, Ates K (2013). The impact of octreotide in experimental proliferative vitreoretinopathy. Indian J Ophthalmol.

[126] Amato R, Giannaccini M, Dal Monte M (2020). Association of the somatostatin analog octreotide with magnetic nanoparticles for intraocular delivery: a possible approach for the treatment of diabetic retinopathy. Front Bioeng Biotechnol.

[127] Escudier B, Bellmunt J, Négrier S (2010). Phase III trial of bevacizumab plus interferon alfa-2a in patients with metastatic renal cell carcinoma (AVOREN): final analysis of overall survival. J Clin Oncol.

[128] Medhat E, Esmat G, Hamza E (2016). Ophthalmological side effects of interferon therapy of chronic hepatitis C. Hepatobiliary Surg Nutr.

[129] Luo WX, He JP, Li X, Liu JY (2015). Neoadjuvant chemotherapy with cetuximab for locally advanced penile cancer. J Cancer Res Ther.

[130] Foerster CG, Cursiefen C, Kruse FE (2008). Persisting corneal erosion under cetuximab (Erbitux) treatment (epidermal growth factor receptor antibody). Cornea.

[131] Melichar B, Nemcová I (2007). Eye complications of cetuximab therapy. Eur J Cancer Care (Engl).

[132] Specenier P, Koppen C, Vermorken JB (2007). Diffuse punctate keratitis in a patient treated with cetuximab as monotherapy. Ann Oncol.

